# Comparative Study between Quantitative Digital Image Analysis and Fluorescence In Situ Hybridization of Breast Cancer Equivocal HER2 2+ Cases

**DOI:** 10.1155/2014/608254

**Published:** 2014-12-11

**Authors:** E. Ayad, M. Mansy, D. Elwi, M. Salem, M. Salama, K. Kayser

**Affiliations:** ^1^Pathology Department, Faculty of Medicine, Cairo University, Cairo, Egypt; ^2^Pathology Department, University of Utah and ARUP Reference Lab, Salt Lake City, UT, USA; ^3^Pathology Department, Humboldt University, Berlin, Germany

## Background

Optimization of workflow for breast cancer samples with equivocal HER2/neu score 2+ results in routine practice remains to be a central focus of the ongoing efforts to assess HER2 status. According to the College of American Pathologists/American Society of Clinical Oncology guidelines equivocal HER2/neu score 2+ cases are subject for further testing, usually by fluorescence in situ hybridization (FISH) investigations. It still remains on open question, whether quantitative digital image analysis of HER2 immunohistochemically (IHC) stained slides can assist in further refining the HER2 score 2+.

## Aim of This Work

The aim of this work is to assess utility of quantitative digital analysis of IHC stained slides and compare its performance to fluorescence in situ hybridization in cases of breast cancer with equivocal HER2 score 2+.

## Material and Methods

Sixty specimens previously (interactively) diagnosed as breast cancer, represented the study population. Her2 stained slides were scored. Cases with HER2/new score of 2++ were digitally scanned by iScan (Produced by BioImagene (Now Roche-Ventana)). The IHC signals of HER2 were measured using an automated image analyzing system (MECES, http://www.Diagnomx.eu/meces). Contemporary new cuts were prepared for FISH examination.

## Results

Three out of the fifteen cases with equivocal HER2 score 2+ turned out to be positive (3+) by quantitative digital analysis, and 12 were found to be negative in FISH too. Two of these three positive cases proved to be positive with FISH, and only one was negative.

## Conclusions

Quantitative digital analysis is highly sensitive and relatively specific when compared to FISH in detecting HER2/neu overexpression. Therefore, it represents a potential reliable substitute for FISH in breast cancer cases which desire further refinement of equivocal IHC results.

